# Efficacy of *liriope platyphylla* extract for improving respiratory functions: Study protocol for a randomized, double-blinded, placebo-controlled pilot trial

**DOI:** 10.1097/MD.0000000000028452

**Published:** 2022-02-11

**Authors:** Ga Hyeon Jung, Chae Hyun Park, Hyun Lee, Jae Hui Kang

**Affiliations:** Department of Acupuncture & Moxibustion Medicine, College of Korean Medicine, Daejeon University, Daejeon, South Korea.

**Keywords:** cough, *liriope platyphylla*, protocol, randomized controlled trial, respiratory function, sputum

## Abstract

**Background:**

Respiratory disease has emerged as a global issue due to COVID-19. In particular, there has been an increased frequency of occurrence of symptoms such as cough, sputum, and dyspnea, which commonly accompany chronic obstructive pulmonary disease (COPD). *Liriope platyphylla* (LP) extract is known to improve respiratory function. LP extract ameliorates the symptoms commonly seen in bronchitis, asthma, and COPD and enhances immunity, as it has anti-inflammatory properties. In a previous study in rats, LP effectively improved respiratory inflammation levels. However, few randomized controlled trials have verified the effects of LP in respiratory disease and there have been no studies to determine the appropriate dose and duration to make it a more convenient functional health product. Based on previous studies, we would like to proceed with this clinical trial under the assumption that LP will help improve respiratory function and produce anti-inflammatory effects.

**Methods:**

This will be a single-center, randomized, double-blind, placebo-controlled pilot trial. Participants will randomly be allocated to receive either 1000 mg LP or placebo. The total duration of the clinical trial will be 4 to 6 weeks. A follow-up assessment will be conducted 4 weeks after screening, and the effect and safety of LP application will be assessed at this second visit. The primary outcome will be the breathlessness, cough, and sputum scale score. Secondary outcomes will include pulmonary function, clinical symptoms of cough and sputum (reported through a questionnaire), changes in immune cells, changes in immune factors used to analyze allergic inflammation in bronchi, antioxidant enzyme activity, nitric oxide level, and COPD assessment test score.

**Discussion:**

This study has limited inclusion and exclusion criteria and the intervention will be well-controlled. This will be the first randomized controlled trial to assess the efficacy and safety of LP extract in adults with cough and sputum. This study will provide insight into the mechanisms of the anti-inflammatory effects and improvement of respiratory function of LP.

## 1 Introduction

Respiratory disease has emerged as a global issue due to COVID-19, which has been prevalent since the end of 2019. In Korea, even before the onset of the COVID-19 pandemic, people were wary of respiratory diseases caused by fine dust. Among air pollutants, fine dust has recently been recognized as the most serious threat to human health.^[1]^ Fine dust affects the respiratory system in particular, as the small size of the particles allows for deposition in the lower bronchial tubes and parenchyma of the lungs, resulting in decreased lung function and increased respiratory symptoms.^[2]^

Bronchial inflammation resulting from fine dust exposure is the most common respiratory disease.^[3]^ The main respiratory symptoms are cough, sputum, shortness of breath, wheezing, and chest discomfort.^[4]^

Accordingly, a need for effective health functional food and medicine products and complementary medicine for respiratory diseases has emerged, and there has been increased interest in and development of products. The monocotyle flowering plant *Liriope platyphylla* (LP) is known to have antioxidant and anti-inflammatory effects^[5,6]^ and immunomodulatory abilities^[7]^ and has been widely used for lung and respiratory diseases. In particular, it is effective for treating bronchitis, pharyngitis, and asthma accompanied by dry cough.^[8,9]^

In this study, LP will be used as a raw material to derive medicine from a natural herbal supplement. The effects of LP on respiratory function, improvement of symptom, and the alleviation of inflammation will be tested.

The following protocol has been designed to verify the safety and efficacy of LP in treating respiratory system symptoms using a single-center, randomized, controlled, and double-blind clinical trial.

## 2 Methods

### 2.1 Study design

We designed a double-blind, single-center, randomized clinical trial to investigate the efficacy of LP in improving respiratory function. A total of 22 participants with respiratory symptoms (cough, sputum) will be recruited from outpatients at the Daejeon University Cheonan Korean Medicine Hospital (DUCKMH) through advertisements posted on bulletin boards at hospitals. Recruitment commenced in July 2021, and the trial is expected to end in December 2021. All participants will receive a full written explanation of the study protocol and an informed consent form.

Pulmonary function tests will be performed for those with respiratory symptoms, including cough or sputum, based on history or questionnaires. Additionally, blood tests for liver function, inflammation-related cells, and complete blood count will be performed.

A total of 22 participants who meet the eligibility criteria for this study will be randomly assigned in a 1:1 ratio to the treatment group (1000 mg LP) or control group (placebo). The intervention will begin within 2 weeks of the screening visit.

**Figure 1:**
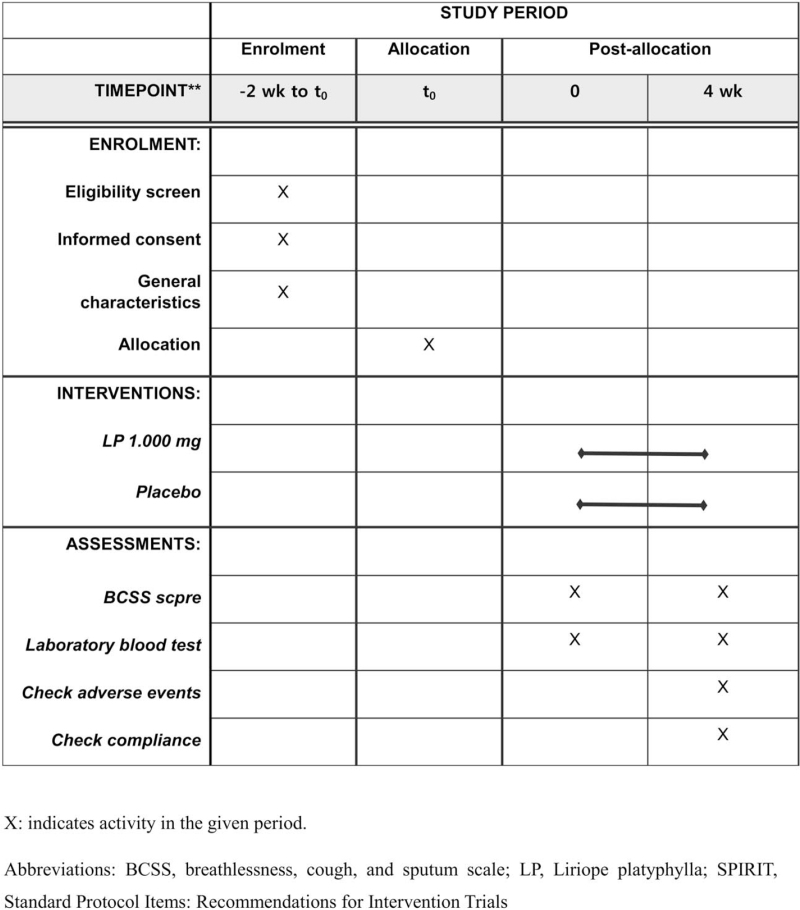
SPIRIT checklist showing the schedule of enrollment, assessments, and allocation for each subject in the *Liriope platyphylla* extract pilot clinical trial protocol.

Based on the study group they are randomized into, the participants will be administered either 1000 mg LP or placebo for 4 weeks. The total duration of the clinical trial will be 4 to 6 weeks, and the efficacy and safety will be assessed at the second visit. A flowchart of the study is shown in Figure 1 and Table 1.

**Table 1:** Clinical trial process for the *liriope platyphylla* extract clinical trial protocol.

Item	Screening (–2 wk to 0 d)	Visit 1 (baseline visit) (0 d)	Visit 2 (trial end) (4 wk ± 5d)
Prepare a written consent form	√		
Evaluate inclusion/exclusion criteria	√		
Examine subject information	√		
Assign screening number	√		
Assign random numbers to subjects		√	
Investigate medical history and medication history	√	√	
Assess vital signs	√	√	√
Conduct clinical laboratory blood test	√	√	√
Conduct pregnancy test	√		
Distribute experimental/control foods		√	
Measure effectiveness-evaluation variables		√	√
Check adverse events			√
Identify concomitant drugs			√
Check compliance			√
Confirm cancellation and withdrawal criteria		√	√

### 2.2 Inclusion and exclusion criteria

The inclusion criteria are as follows:

1. age 19 to 80 years;
2. persistent respiratory symptoms (cough, sputum);
3. forced expiratory volume in 1 second (FEV1)/forced vital capacity (FVC) level, above 70%; and
4. provision of written consent to participate in the trial.

The exclusion criteria are as follows:

1. past or present history of respiratory diseases (respiratory diseases involving damaged lung parenchyma, such as tuberculosis or bronchiectasis; inflammatory lesions in the lung parenchyma due to pneumonia or tuberculosis; undergoing treatment for bronchial asthma, COPD, influenza, pneumonia, or lung cancer; acute or chronic bronchitis with breathlessness, cough, and sputum scale score (BCSS) >9 and persistent cough or sputum for at least 3 months per year for 2 consecutive years);
2. uncontrolled cardiovascular disease;
3. use of systemic corticosteroids, immunosuppressants, health functional foods, or medicine to control the respiratory system in the 4 weeks prior to the study;
4. serum creatinine > 2 mg/dL, or on dialysis for chronic kidney failure;
5. total bilirubin ≥2 mg/dL, or aspartate aminotransferase or alanine aminotransferase blood levels ≥3 times the normal upper reference limits;
6. uncontrolled psychological disease or alcoholism;
7. participation in interventions in other clinical trials within 1 month of the screening visit;
8. pregnant or breastfeeding; and
9. unsuitability for this study (as determined by the investigator) based on overall health status.

### 2.3 Dosage calculation

We have previously demonstrated in both *in vitro* and *in vivo* experiments that LP improves inflammation and shows no toxic effects. To determine the minimum effective concentration, LP was administered at 200 mg/kg to mice for 14 days. Based on the United States Food and Drug Administration guidelines for calculating the effective dose according to body surface area in humans, the effective dose in the animal tests was used to calculate the effective dose in terms of the human body. The human dose was set at 960 mg. For this study, the dose was set at 1000 mg in consideration of the ease of production and intake.

### 2.4 Sample size calculation

This study is a pilot trial to explore the effect of LP on the primary and secondary outcome measures selected for a minimum sample size, and to investigate the sample size calculation basis and feasibility for future clinical trials. According to Whitehead et al,^[10]^ when the power is considered to be 80% and effect size is considered moderate, in a preliminary parallel-designed study the empirically optimal sample size is 10 persons per group. As this current study plan includes 2 visits within 4 weeks, high compliance is expected, which suggests a calculated dropout rate of 5%. Therefore, we designed a pilot study involving 22 participants.

### 2.5 Randomization and blinding procedures

Randomization will be performed using the block randomization method in the experimental and control groups at a ratio of 1:1. The size of the block will be set randomly by a statistician. Subjects who meet all the registration criteria will be randomly assigned a randomization identification code (e.g., DLB-R-001, DLB-R-002, …, DLB-R-022) in the order generated by a computer-randomized program. The randomization number will be given to each participant and will indicate whether 1000 mg LP or placebo is to be given. As this pilot study was designed to be double-blinded, the participants, researchers, and assessors collecting the data will be blinded to the group allocation. Information on the intervention assignment will be stored in the statistical department. The randomization code will be placed in an opaque envelope and stored in the hospital. With the exception of disclosure of an individual patient's status in the case of a serious adverse event, randomization and blinding will not be disclosed to researchers until the end of the trial.

### 2.6 Intervention

All eligible participants will receive treatment according to their allocated group (LP or placebo) during the 4-week treatment period. To maintain the double-blinded nature of the study, the 1000 mg LP and placebo will be manufactured to be indistinguishable in appearance by D&L Biochem (Chungju, Republic of Korea). One capsule will be taken orally twice a day. Any other treatment received during their participation in the trial, functional health foods taken, or additional therapies will be considered to be combination therapy. If coadministration is necessary, the administration details will be recorded in the case report form and treatment chart, and the existing therapy will remain unchanged. The participants will be evaluated after 4 weeks.

Concomitant drug administration will however be minimized during trial participation, and will be allowed based on the judgment of the principal investigator when it is deemed inconsequential to the trial. If a drug directly affects immune function, that participant will be withdrawn from the trial.

### 2.7 Outcome measures

**Figure 2:**
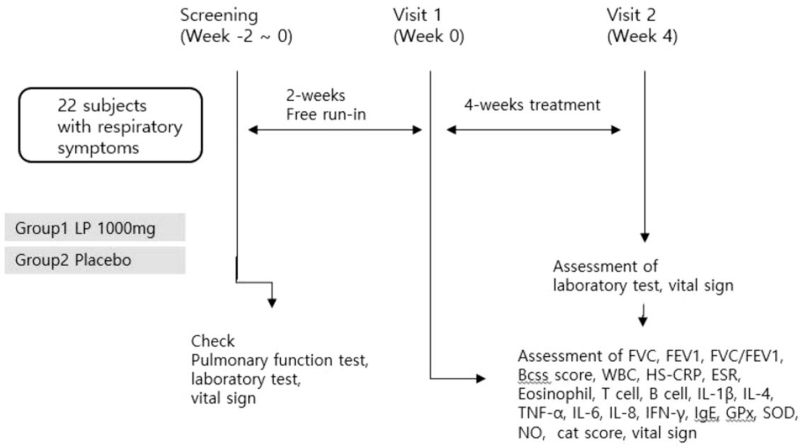
Study design for LP clinical trial. The treatment or control groups will be administered. LP 1000 mg or placebo. The subjects will receive LP 1000 mg or placebo for 4 weeks and will visit 2 times.

The primary outcome is the BCSS score, which will be used to assess respiratory symptoms, such as dyspnea, cough, and sputum. The secondary outcomes are the pulmonary function test (FVC, FEV1, FEV1/FVC), the clinical symptoms of cough and sputum (assessed through a questionnaire), high-sensitivity C-reactive protein level, erythrocyte sedimentation rate, white blood cell count, eosinophil, T cell, B cell, and immune factors (interleukin (IL)-1β, IL-4, tumor necrosis factor (TNF)-α, IL-6, IL-8, interferon (IFN)-γ, immunoglobulin (Ig)E) to analyze allergic inflammation in the bronchi, antioxidants (glutathione peroxidase, superoxide dismutase, nitric oxide) to analyze bronchial peroxide expression levels, and COPD assessment test score. All outcome measures will be assessed at the first and second visits. To ensure the safety of the participant, we will conduct laboratory tests, such as liver function, complete blood count, and electrolytes, and evaluate vital signs at every visit. Safety assessments will be conducted during the second visit. The participants’ data will be anonymized and coded using a specific program (Fig. 2).

### 2.8 Data collection

During screening, the participants will complete a questionnaire regarding their sociodemographic characteristics and provide a history of respiratory disease within the past 2 years, their medical history of the last 3 months, and their drug history of the previous 4 weeks. Lung function tests, laboratory tests for liver function, complete blood counts, immune function assessment, and inflammatory factor assessments will also be conducted. Personal information and data collected during the screening period will be managed by the hospital. The final dataset will be accessible to statisticians and key investigators. The results of this study will be published in a peer-reviewed journal.

Personal information will be collected during screening. The investigator will process the data, including personal information, under the clinical study protocol approved by the institutional review board (IRB). The participants’ names will remain confidential and participants will be identified only by their assigned randomized number.

The investigator will secure the data, including personal information, in locked cabinets. Access to the data will be restricted. All data will be handed to the storage manager after the clinical trial results have been reported, and the data will be preserved for 3 years from the date of completion of the trial.

### 2.9 Statistical analysis

Statistical analyses will be performed using the principle of the full analysis set (FAS). For the intention-to-treat analysis, missing values will be analyzed as last observation carried forward. The baseline values before treatment and the changes in BCSS scores after 4 weeks of treatment will be measured for the experimental and control groups. To test the mean rank sum of BCSS changes after the 4 weeks compared to baseline between the 2 groups, the Mann–Whitney-Wilcoxon rank sum test will be performed. The treatment difference between the treatment and control groups will be estimated using the Hodges-Lehmann method, and a 95% nonparametric confidence interval will be presented. The confidence level will be set to 5%. All statistical analyses will be performed using SPSS Statistics for Windows version 21.0 (IBM Corp., Armonk, NY).

Descriptive statistics, such as the number of participants, and the mean, standard deviation, minimum, median, and maximum of data, will be presented for each visit, as well as changes from baseline at the final evaluation for continuous data, clinical laboratory tests, and vital signs. No intermediate analyses will be performed.

Descriptive statistics for demographic data and clinical history data for the FAS group will be presented. Continuous data will be tested using *t*-tests or Wilcoxon's rank sum tests. Categorical data will be analyzed using the chi-square test or Fisher exact test.

The safety assessment calculates the rate of adverse events. Continuous data such as comparisons between groups, individual laboratory test results, and biomarkers will be analyzed using paired *t*-tests or *t*-tests with 95% confidence intervals within the group to determine if there are any changes compared to the baseline. If the assumption of normality is not met, nonparametric statistical analyses (Wilcoxon signed rank test or Wilcoxon rank sum test) will be performed. Categorical data will provide frequencies and ratios for each category. The differences between groups will be analyzed using the chi-square test and Fisher's exact test.

### 2.10 Withdrawal and dropout

If participants do not meet the inclusion or exclusion criteria (for screening only), withdraw their consent, or if the participant's continued participation is judged to be inappropriate, participants will be excluded from the study. In addition, participants may be excluded from the study if there is a major violation of the human application test protocol or non-observation. Those who have taken or need to take medicines or health functional foods that may affect this clinical trial may be excluded from the study; if a participant becomes pregnant during the human application test period, the participant may be withdrawn. The researcher will record the reason for the treatment interruption and whether each participant completed the study.

### 2.11 Safety

The occurrence of side effects will be assessed at each visit. Subjects will be monitored for undesirable and unintended symptoms, signs, and diseases. All adverse events will be classified and evaluated as mild, moderate, or severe. If a serious adverse event occurs, it will be reported to the IRB within 24 hours of identification and the participant will be required to withdraw. The number and ratio of participants who experience adverse events will be recorded.

### 2.12 Ethics

The study design is in accordance with the Helsinki Declaration and the Korean Clinical Practice Guidelines and was approved by the Korean IRB of DUCKMH (approval number DJUMC-2021-BM-02-1). This study protocol was registered with the Korean National Clinical Research Information Service (CRIS) (CRIS-KCT0006426). Any participant in this study may withdraw consent or voluntarily cease to participate at any time for any reason.

## 3 Discussion

The lungs and respiratory system prevent various diseases by activating the immune system when viruses invade.^[11]^ However, because they are connected to the outside environment, inflammation occurs easily, leading to symptoms.^[12]^ The most common symptoms are cough, sputum, and shortness of breath; exposure to external fine dust, viruses, and other air pollutants is considered a risk factor.^[4]^

Respiratory diseases such as bronchitis, asthma, and COPD are among the most common diseases, and respiratory symptoms are closely related to discomfort in daily life.^[3]^ Therefore, interest in easily accessible health functional products and complementary medicine has increased. Studies are being actively conducted to discover immune-enhancing substances or extracts isolated from natural herbal supplements.^[13]^

As a traditional Asian herbal medicine, LP has long been used for the treatment of asthma, as well as bronchial and lung inflammation,^[14,15]^ and it is said to act as a defense mechanism against fine dust and respiratory diseases.^[16]^

There have been previous studies on the actions and effects of LP. Kim et al reported that LP generated an immunomodulatory effect through T cells and the promotion of INF-γ secretion.^[17]^ Ryu et al reported the effects of LP on the secretion of cytokines, eosinophils, and IgE in relation to asthma.^[16,18]^ Kim et al reported the effects of LP on immune cells in a rat asthma model.^[19]^ LP has also been shown to potently inhibit airway inflammation and hyperresponsiveness in a murine model of asthma by modulating the relationship between Th1/Th2 cytokine imbalance.^[20]^ Most recently, LP has been noted to suppress the increased inflammatory cytokines in lung phagocytic cells or epithelial cells caused by fine dust in a dose-dependent manner.^[5,21]^

The proposed study will be the first at this stage to be conducted to determine the appropriate dosage and duration of LP administration. Although our sample size will be small and biases may result, this study will provide valuable insights into the underlying mechanisms of the anti-inflammatory effects and improvement of respiratory function caused by LP extract and will lay the groundwork for further studies.

## Author contributions

**Project administration:** Jae Hui Kang.

**Supervision:** Hyun Lee, Jae Hui Kang.

**Writing – original draft:** Chae Hyun Park, Ga Hyeon Jung.

**Writing – review & editing:** Chae Hyun Park, Ga Hyeon Jung.
